# Accessibility at a primed distal *Fshb-Kcna4* super-enhancer is facilitated by Foxl2 during gonadotrope differentiation

**DOI:** 10.1210/endocr/bqag028

**Published:** 2026-03-13

**Authors:** Tal Refael, Gil Golan, Daniella Darsa, Lilach Pnueli, Probir Chakravarty, Karine Rizzoti, Philippa Melamed

**Affiliations:** Faculty of Biology, Technion-Israel Institute of Technology, Haifa 32000, Israel; Faculty of Biology, Technion-Israel Institute of Technology, Haifa 32000, Israel; Faculty of Biology, Technion-Israel Institute of Technology, Haifa 32000, Israel; Faculty of Biology, Technion-Israel Institute of Technology, Haifa 32000, Israel; Bioinformatics Core, The Francis Crick Institute, London NW1 1AT, UK; Laboratory of Stem Cell Biology and Developmental Genetics, The Francis Crick Institute, London NW1 1AT, UK; Centre for Endocrinology, William Harvey Research Institute, Faculty of Medicine and Dentistry, Queen Mary University of London, London EC1M 6BQ, UK; Faculty of Biology, Technion-Israel Institute of Technology, Haifa 32000, Israel

**Keywords:** Fshb, Kcna4, gonadotrope, enhancer, transcription, Foxl2

## Abstract

A large “gene desert” located far upstream from *Fshb* and *Kcna4* contains several gonadotrope-specific accessible chromatin sites that were seen in chromatin conformation capture to make distinct contacts with both genes. Expression of *Fshb* and *Kcna4* was strongly inhibited by JQ-1, which represses super-enhancer activity, and the region displays super-enhancer characteristics. The sites of open chromatin were seen, in chromatin immunoprecipitation, to bind Brd4 and Med1, most notably at a site −67 kb from the *Fshb* gene, as well as binding Ctcf further upstream (−123 kb), all of which were increased following activin exposure. The locus is transcribed to chromatin-associated long noncoding RNAs whose levels correlate with *Fshb* and *Kcna4* mRNA levels in vivo and in cultured gonadotrope cells, indicating coordinated regulation. CRISPR interference confirmed distinct functions for each element and, together with the chromatin conformation capture data, indicate that the −67 kb locus mediates basal and activin-stimulated *Fshb* expression, whereas the site at −59 kb contributes to activin-stimulation of both genes. Single-cell multiomics revealed that the −67 kb locus is accessible in pituitary stem cells and throughout gonadotrope differentiation, preceding opening of the *Fshb* promoter, although it is closed in other differentiated cell types, suggesting a gonadotrope-specific factor that keeps it open at this stage. Foxl2 was found to bind this element, contributes to maintaining its chromatin accessibility, and recruits Supt16h, a component of the Facilitates Active Chromatin Transcription histone chaperone complex. These findings define a distal, Foxl2-bound super-enhancer that regulates *Fshb* transcription and shapes the gonadotrope regulatory landscape.

Distal *cis*-regulatory elements are major determinants of tissue-specific temporal and signal-responsive gene expression, but can be challenging to study because of the linear distances from their target genes and unclear modes of action. They are frequently transcribed into long noncoding RNAs (lncRNAs) and enhancer RNAs (eRNAs), and typically engage in long-range interactions with their target promoters, recruiting transcriptional regulators and chromatin-modifying complexes to establish a permissive chromatin landscape (1-9). In some loci, enhancers occur in clusters known as super-enhancers (10, 11). These are often located within large gene deserts and characterized by a particularly dense occupancy of regulatory proteins and abundant noncoding transcription that are thought to promote robust activation of lineage-defining genes through formation of regulatory hubs (7, 10, 12). These distal elements operate across large genomic distances within the constraints of higher-order chromatin architecture, the topologically associated domains (TADs) demarcated by proteins such as CTCF and cohesin (13, 14).

Not only are the molecular mechanisms through which distal elements affect transcription of their targets incompletely understood, but most genes are likely regulated by many such elements (15). These loci may act additively, synergistically or antagonistically, and some of the individual regulatory regions may well influence several different genes (9, 16-18). Although commonly identified based on chromatin features, only a fraction of these putative regulatory elements have been functionally validated, and there remains a need to characterize and define them according to their biological activity in the appropriate physiological context (15). This is essential for understanding how sequence variation in noncoding genomic regions underlies altered phenotypes and disease.

We previously identified distal enhancers that are critical for transcription of the pituitary gonadotropin subunit *Cga* and *Lhb* genes. The *Cga* enhancer directs tissue-specific expression (19, 20) and produces an eRNA essential for establishing permissive chromatin and enabling transcription at this locus (5). Similarly, an upstream *Lhb* enhancer generates a regulatory eRNA, and a distinct lncRNA that contributes to *Lhb* splicing (21). Previous work described enhancer activity for the murine *Fshb* gene by a conserved site 17 kb upstream of the gene that is homologous to the human sequence which contains a fertility-associated single nucleotide polymorphism (22, 23). However, despite exhibiting activity in vivo, the deletion of this DNA sequence in vivo had no apparent effect on fertility (24). The *Fshb* upstream nearest neighbor gene is located ∼230 kb away, and we hypothesized in this study that this large “gene desert” harbors additional regulatory elements that act to shape *Fshb* expression during gonadotrope differentiation and establish and maintain gonadotrope identity.

Although integration of ATAC-seq chromatin accessibility with simultaneous measurement of transcriptional output in single-cell multiomics offers a powerful means to identify and dissect putative enhancer function, models for gonadotrope differentiation have been lacking. Recently, however, we demonstrated that neonatal pituitary stem cells undergo a wave of differentiation into gonadotropes (25), providing a unique window to study these events during establishment of this lineage. Here, we demonstrate a function for a distal super-enhancer element that not only regulates *Fshb* expression but also coordinates that of its neighboring gene, *Kcna4,* and is kept open early in gonadotrope lineage differentiation by Foxl2.

## Materials and methods

### Cell culture, treatments, and transfections

The murine (male and female, respectively) gonadotrope-derived αT3-1 and LβT2 cell lines (gift from P. Mellon, University of California, San Diego) were cultured as described (26) and are authenticated routinely (multiple times each year), based on gonadotropin gene expression levels. As noted, they were treated with vehicle alone (nontreated controls), Activin A (5 nM; #SRP6057, Sigma or Peprotech #120-14P, Gibco) or JQ-1 (0.25 or 1 µM; #CAY-11187-1, Cayman Chemicals). CRISPRi was carried out as previously (21) following stable transfections of dCas9-KRAB (Addgene #50919) and gRNA oligos (Table S1 (27)).

### Chromatin conformation capture assay

Chromatin conformation capture (3C) assay was carried out in LβT2 cells, as described previously (5). PCR was performed using 1 μL of the library per 50-μL reaction. The primers comprised nested forward primers targeting −261 and −210 bp from the *Fshb* transcriptional start site (TSS), and −533 and −487 bp from the *Kcna4* TSS, with sets of primers targeting various upstream sites as shown and detailed in Table S2 (27). The first round of PCR was at 55 °C for 35 cycles and the second round at 55 °C for 25 cycles. Amplicons were verified by sequencing.

### RNA extraction and real-time quantitative PCR

Total RNA was extracted using TRIzol (Ambion), treated with DNase and purified using R1014 RNA clean and concentrator-5 kit (Zymo Research). The cDNA was synthesized using qScript cDNA Synthesis Kit (Quanta Biosciences), or High Capacity cDNA Reverse Transcription Kit (Applied Biosystems) according to the manufacturer's instructions. Real-time quantitative PCR (qPCR) was carried out using the PerfeCTa SYBR Green FastMix (Quanta Biosciences) or SYBR green blue mix Hi-ROX (PCR Biosystems), using primers listed in Table S2 (27). Amplicon levels were quantified using standard curves and normalized to *Rplp0*, unless noted otherwise.

### Chromatin-associated RNA and sequencing

Cellular fractionation and isolation of chromatin associated RNA was performed according to a published protocol (28). Briefly, LβT2 cells grown on 10-cm plates at 70% to 90% confluency were washed twice with PBS, and then collected into an Eppendorf tube, pelleted (845*g*, 4 minutes, 4 °C), and resuspended with 400 μL Igepal buffer (10 mM Tris pH7.4, 150 mM NaCl, 0.15% Igepal CA-630, Sigma Aldrich) by pipetting up and down 3 to 5 times. The sample was incubated on ice for 5 minutes, laid gently on 1 mL of sucrose cushion (10 mM Tris pH 7.4, 150 mM NaCl, 24% sucrose) and centrifuged at 1000*g* for 10 minutes at 4 °C. The top 400 μL comprised the cytosolic fraction. The rest of the supernatant was discarded, and the pellet washed with 1 mL PBS with 0.5 mM EDTA, and centrifuged for 5 seconds at 1000*g*. The supernatant was discarded and the pellet resuspended with 250 μL of glycerol buffer (20 mM Tris pH 7.4, 75 mM NaCl, 0.5 mM EDTA, 50% glycerol), to which 250 μL of urea buffer was added (10 mM Tris pH 7.4, 1 M urea, 0.3 M NaCl, 7.5 mM MgCl2, 0.2 mM EDTA, 1% Igepal CA-630). The sample was vortexed for 4 seconds, incubated for 2 minutes on ice and centrifuged at 13,000*g* for 2 minutes at 4 °C. The supernatant was collected as the nuclear fraction and the pellet as the chromatin fraction. All buffers contained RNAsin to inhibit RNAse. For RNA extraction, 3 volumes of Trizol were added, and the Trizol protocol followed until phase separation. The upper phase was then taken for cleanup with DNAse (RNA Clean & Concentrator Kits, Zymo Research R1014).

RNA libraries were created for the chromatin associated and cytoplasmic fractions and sequenced using the Illumina HiSeq 2500 at the Technion Genome Center. Subsequent analysis included mapping using Tophat 2, counting with HTseq, library quality control in FASTQC, and differential expression analysis with the DESeq2 R package. The reference genome used in the analysis was the *Mus musculus* mm10 (GRCm38.p5).

### Animal surgery and tissue harvest

Pituitaries were harvested from male and female C57BL6/J mice of various ages, before and after puberty, and collected to 1 mL Trizol for RNA extraction. Ovariectomy was performed as reported previously (29), and mice sacrificed 14 days after operation; for sham-operated female mice, this was during estrus at least 14 days postsurgery. The pituitaries were then harvested, homogenized, and RNA was extracted as described previously. The mice were held at the Technion preclinical facility SPF-wing, and handled after protocol approval (IL209-1221), in accordance with Technion IACUC guidelines.

### Foxl2 knockout by CRISPR editing

Candidate gRNAs (Table S1 (27)) were designed using Benchling (https://www.benchling.com), chosen according to efficiency and specificity scores provided by Benchling and CHOPCHOP (https://chopchop.cbu.uib.no). The annealed gRNAs were inserted into pSpCas9(BB)-2A-GFP (PX458, Addgene #48138) cut with *BbsI*, and verified by sequencing. The plasmids (1 μg) were transfected to a 35-mm dish of αT3-1 cells using Polyjet reagent (SignaGen) according to the manufacturer's instructions and, 24 hours later, the GFP-positive cells were collected using a BD ARIA cell sorter at 4 °C with a 100-µm nozzle, flow rate 1, and single cell mode. Two or 4 GFP-positive cells were deposited into each well of 96-well plates preloaded with 100 µL of culture medium. The plates were incubated at 37 °C with 5% CO_2_ and monitored for colony formation, and only wells with a single clone remaining were selected for further expansion. Genomic DNA was extracted from the expanded clones using the D4068 Quick-DNA Miniprep Plus Kit (Zymo Research). To screen for mutations, the gRNA target region was PCR-amplified from both αT3-1 WT and clonal DNA using KAPA HiFi HotStart polymerase (primers: forward 5′-ATGGCCAGAGGCTGACTTC-3′ and reverse 5′-GCCAGGTAGCCATAGCCAT-3′). To detect insertions or deletions, equal amounts of wild-type (WT) and clonal PCR fragments were mixed to allow for heteroduplex formation. This mixture was then analyzed using the Alt-R Genome Editing Detection Kit (T7E1 assay # 1075931, IDT) according to the manufacturer's instructions for T7 Endonuclease I digestion. Clones exhibiting cleavage products indicative of editing were validated by Sanger sequencing and western blot analysis. The clone used here was the result of a homozygous frameshift introducing a stop codon after the first 81 amino acids.

### Chromatin immunoprecipitation

Chromatin immunoprecipitation (ChIP) experiments for H3 (Ab1791 RRID:AB_302613), Ctcf (Ab128873 RRID:AB_11144295), Brd4 (Ab243862 RRID:AB_3096484), Med1 (Ab64965 RRID:AB_1142301), IgG control (Ab6721 RRID:AB_955447; all from Abcam), Foxl2 (NB100-1277 RRID:AB_2106188; Novus Biologicals), and SUPT16H (#12191, RRID:AB_2732025, Cell Signalling) were carried out in formaldehyde cross-linked LβT2, αT3-1 WT, and Foxl2 KO cells. For the H3 ChIP, cells were sonicated using 60 × 15″ on, 10″ off pulses, producing an average of 150- to 200-bp fragments, otherwise sonication was with 10 × 15″ on, 10″ off pulses, producing an average of 500-bp DNA fragments, both as described (5). Real-time qPCR (primers in Table S2 (27)) amplified specific regions in the IP and input samples, for normalization.

### Coimmunoprecipitation

LβT2 cells (1 × 10^7^ cells in 12 × 100-mm plates) were plated and fast-forward transfected using Fugene 4k (Promega; # E5911) with either HA-Foxl2 (Foxl2 cloned into pxj40-HA) for N-terminal HA tagging or with pEGFP-N1 (Clontech) as control. On the following day, the media were replaced, as noted, to include 5 nM Activin A (Peprotech #120-14P, Gibco) and after 24 hours, the cells were collected and rinsed twice in PBS. The pellet from each plate was suspended in 1 mL of hypotonic buffer with protease and phosphatase inhibitors (25 mM HEPES pH 7.9, 1.5 mM MgCl2, 10 mM KCl, NP40 0.1%, 1 mM DTT, PMSF 1 mM, Roche Complete Protease Inhibitor Cocktail [# 11873580001], 1 mM NaF, 1 mM sodium vanadate, 1 mM B glycerol phosphate). After 10 minutes on ice, they were centrifuged at 2348*g* for 5 minutes at 4 °C, and the pellet resuspended in 500 µL NP40 buffer with protease and phosphatase inhibitors (50 mM Tris pH 7.4, 150 mM NaCl2, 1 mM MgCl2, NP40 1%, PMSF 1 mM, Roche Complete Protease Inhibitors, 1 mM NaF, 1 mM sodium vanadate, 1 mM B glycerol phosphate) with 5 µL of Benzonase (#70664-3, Sigma), for 1 hour incubation on a roller at 4 °C. After centrifugation for 5 minutes at 9391*g* at 4 °C, the cleared supernatant was taken for coimmunoprecipitation, using 3 µg of HA antibody (Abcam, # ab9110, RRID:AB_307019) for 1 hour incubation on a roller at 4 °C, before addition of 20 µL protein G (#10004D, Invitrogen washed twice with PBS). After incubation overnight on a roller at 4 °C, the IP was washed 3 times with NP40 buffer and eluted for 40 minutes at room temperature with 50 µL of HA peptide (#11666975001, Sigma; 150 ng/µL) in PBS. The eluted protein was resolved ∼3 cm into a 12.5% SDS-PAGE gel and excised. The proteins were then trypsinized and analyzed by tandem mass spectrometry Data-Dependent Acquisition using Q Exactive HFX MS (Thermo), and data analyzed and quantified by MaxQuant 2.6 software, all at the Technion Smoler Proteomics Center, as previously (21).

### Neonatal pituitary multiomics

All animal experiments were approved under the UK Animals (Scientific Procedures) Act 1986 under the project licence PP8826065 and by the Francis Crick Animal Welfare and Ethical Review Body (AWERB). *Sox9^iresGFP/+^* (*Sox9^tm1Haak^*) (30) mice were maintained on a C57Bl6 background.

Pituitaries from P3 *Sox9^iresGFP/+^* pups were dissociated and sorted as described (31) separately for males (8 pups) and females (6 pups). Nuclei were then extracted following the 10X Genomics protocol (https://cdn.10xgenomics.com/image/upload/v1745274099/support-documents/CG000366_DemonstratedProtocol_SingleCellMultiome_Nuclei_EmbMouseBrain__Rev_E.pdf).

The concentration and viability of the single nuclei suspension was measured using acridine orange and propidium iodide, and the Luna-FX7 Automatic Cell Counter. Approximately 20 000 nuclei were transposed, then loaded on Chromium Chip and partitioned in nanoliter scale droplets using the Chromium Controller and Chromium Next GEM Single Cell Reagents (Chromium Next GEM Single Cell Multiome ATAC+ Gene Expression Reagent Kits User Guide, User Guide, CG000338).

A pool of 736 000 10 × barcodes was sampled to index the transposed DNA and cDNA of each individual nucleus separately and uniquely. ATAC and gene expression libraries were generated from the same pool of preamplified transposed DNA/cDNA and sequenced using the Illumina NovaSeq 6000. Sequencing read configurations were 28-10-10-90 (gene expression) and 50-8-24-49 (ATAC). The 10 × barcodes in each library type are used to associate individual reads back to the individual partitions, and thereby to each individual nucleus.

### Bioinformatic analysis

The snATAC-seq data for adult male and female mouse pituitaries were downloaded from the corresponding GEO for each animal separately. A Seurat object was created, using the Signac R package for snATAC-seq data analysis (Signac version: 1.8.0; R version: 4.2.1) (32), and cluster identities were assigned to each cell according to the analysis conducted in the original article (cell identities data provided by F. Ruf-Zamojski). The fragment file was then separated to distinct fragment files (BED files) for each cluster, using the “splitFragments” Signac command. These files were uploaded to “usegalaxy” web platform and converted to BAM files using the “bedtools BED to BAMconverter” (Galaxy Version 2.30.0) tool (33). Finally, BAM files were converted to BigWig files to allow visualization of the chromatin landscape in each cluster in IGV, by the “bamCoverage” (Galaxy Version 3.5.1.0.0) tool (34). Bin size was determined as 1 base, and tracks were normalized to the total number of fragments in each cluster (calculated as the number of entries in each fragment file).

The neonatal pituitary multiomics data, raw reads were aligned to the mm10 transcriptome (version 2020) using CellRanger Arc (version 2.0.0) for RNA expression and ATAC peaks quantification, providing an average of 10 124 linked genes and 46 710 linked peaks for 16 417 cells. Subsequent analyses were performed using the Seurat (version 4) package in R (version 4.1).

For the snRNA-seq expression: cell filtering was conducted based on the total number of unique molecular identifiers, the number of detected genes, and the proportion of mitochondrial gene expression. The percentage of mitochondrial gene expression was defined as the proportion of total gene expression attributed to mitochondrial genes. Cells exhibiting elevated mitochondrial gene expression were identified and removed. Quality control was implemented using the median absolute deviation, and cells with values exceeding 3 median absolute deviations from the median were classified as outliers and excluded from further analysis. Doublets were identified using DoubletFinder (version 2.0.4) and scDblFinder (version1.20.2); cells called Doublets by both methods were removed. PCA decomposition was performed per sample using the first 20 components to construct uniform manifold approximation and projection (UMAP) plots per samples. Male and female samples were integrated using Seurat's rpca integration method to take into account any difference in cellular composition, using the top 3000 variable genes and the first 40 principal components. Clusters were annotated using cell-specific signature genes, with the AddModuleScore function.

For snATAC-seq analysis: the data were processed using the Signac R package (version 1.10.0). Peaks of transposition were identified within cell types using MACS2 (2.2.7.1) implemented in Signac's CallPeaks function. Peaks were linked to TSS using the LinkPeaks function with default parameters. Analysis of the chromatin accessibility within cell type-specific peaks followed the Signac pipeline: normalization by RunTFIDF, variable peak selection with FindTopFeatures, and scaling and decomposition using RunSVD. The “lsi” reduction was used to calculate t-SNE and UMAP reductions using dimensions 2 through 15 and the RunTSNE and RunUMAP functions. Clusters were identified using the FindNeighbors and FindClusters functions at a range of resolutions from 0.2 to 2. Differential accessibility was assessed in unified peaks, defined using the UnifyPeaks function of Signac to implement the reduce function of GenomicRanges (1.56.1), within cell types between conditions using the FindMarkers function, as previously.

Gonadotropes (Cluster 11) were subsetted for downstream analysis. We recalculated Weighted Nearest Neighbors using PCA dimensions 1 through 50 (RNA) and LSI dimensions 2 through 50 (ATAC). Dimensionality reduction was performed via UMAP on the weighted graph, and subclusters were identified using the Smart Local Moving algorithm at a resolution of 0.7, yielding 5 subclusters (11A-11E). Subcluster 11E contained only 10 cells and so was excluded from further analysis. BigWig files were generated as for the adult pituitary snATAC-seq data.

Plots were generated in R-package with ggplot2. Protein binding data is from ReMap, 2022 (via UCSC genome browser: https://genome.ucsc.edu/) which includes analysis of 5505 quality-controlled mouse ChIP-seq experiments from public sources such as GEO and ENCODE, and ChIP-atlas (https://chip-atlas.org).

### Statistical analysis

All data are derived from multiple biological replicates (n-values), each assayed individually. Homogeneity of variance was assessed using an F test, and data were analyzed accordingly using 2-tailed Student *t*-tests to determine statistical significance, defined as *P* < .05 (bolded in figures). For multiple comparisons, ANOVA followed by Tukey's honestly significant difference post hoc test was used. Pearson correlation analyses were performed in R.

## Results

### The “gene desert” between *Fshb* and *Kcna4* contains several sites of gonadotrope-specific accessible chromatin that contact both genes and might compose a regulatory hub

**Figure 1:**
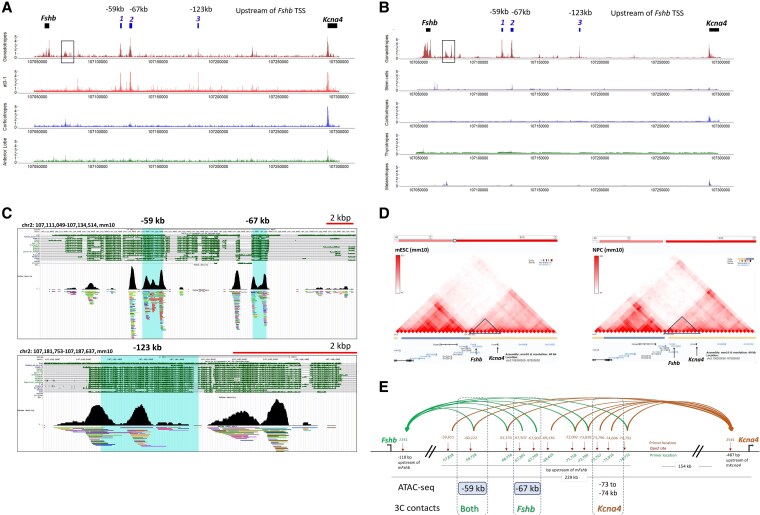
The “gene desert” between *Fshb* and *Kcna4* contains several sites of gonadotrope-specific accessible chromatin which contact both genes and might comprise a regulatory hub. (A, B) ATAC-seq data showing gonadotrope-specific accessible regions of chromatin at the *Fshb-Kcna4* locus, in (A) enriched population of primary gonadotrope cells from adult male mice and the αT3-1 gonadotrope cell line (also shown are data from corticotropes [blue track] and anterior lobe cells after gonadotrope removal [green track; data from (35)); (B) single nuclei from various cell populations of adult male mice pituitaries (data from (36)). The proximal boxed region encompasses the previously reported −17 kb enhancer (22, 23), and 3 more distal peaks of gonadotrope-specific open chromatin are marked. (C) Sequence conservation (green) of the region centered on each of the three more distal sites of open chromatin showing also protein binding from multiple tissues (from ReMap 2022, which includes 5505 mouse ChIP-seq experiments from public sources: GEO and ENCODE) (37); in the UCSC genome browser. (D) *Fshb* and *Kcna4* are located in the same TADs in mouse embryonic stem cells (mESCs; left panel) and neuronal precursor cells (NPCs; right panel; data from (38) analyzed in http://3dgenome.fsm.northwestern.edu/view.php (39)). (E) Interactions (confirmed by sequencing) detected from chromatin conformation capture (3C) in LβT2 gonadotrope cells after *Dpn2* restriction, analyzed by PCR using nested forward primers and those targeting the various upstream regions, as noted. Interactions for *Fshb* are marked in green and for *Kcna4* in brown. See also Figs. S1 and S2 (27).

A large “gene desert” separates *Fshb* from its neighboring gene, *Kcna4,* which is located 230 kb upstream. Initial analysis of ATAC-seq data from male mice (35) revealed that this region contains several loci of open chromatin specifically in murine gonadotropes and the αT3-1 gonadotrope-precursor cell line (Fig. 1A). These include a region ∼17 kb upstream of the *Fshb* TSS gene that was recently reported to act as a transcriptional enhancer (22, 23), and 2 particularly prominent peaks further upstream, at −59 and −67 kb. An accessible site is evident also at −123 kb, though the signal is somewhat greater in the gonadotrope cell line than the primary gonadotrope cells. These same sites of open chromatin are seen in single-nuclei ATAC-seq data from adult mouse pituitaries (36) and are again highly specific to the gonadotrope population (Fig. 1B).

The DNA sequences at these 3 distal sites of open chromatin, particularly at −59 kb and −67 kb, are conserved in mammals and associated with binding of numerous proteins in nonpituitary cell contexts (from ReMap, 2022 (37); Fig. 1C), indicating likely function as a regulatory hub. Although located far from the *Fshb* gene, the entire region, including the upstream *Kcna4* gene, is found in the same TAD in mouse embryonic stem cells and neuronal precursor cells (Fig. 1D) (38, 39), raising the possibility that it might interact with either or both genes. Notable also is that *Kcna4* expression in the pituitary is particularly enriched in gonadotrope cells, though not restricted to this cell type (Fig. S1 (27)).

We therefore performed 3C analysis in the LβT2 gonadotrope cell line to determine if these distal regions are in physical contact with *Fshb* and/or *Kcna4*. The 3C analysis revealed interactions between the −59 kb site with both *Fshb* and *Kcna4*, whereas the site at −67 kb interacted only with *Fshb*. We also found that the region just upstream, between −73 to −74 kb, interacted with *Kcna4* but not *Fshb* (Fig. 1E, Fig. S2 (27)).

### The region has characteristics of a super-enhancer, is associated with chromatin organizational proteins, and transcribed to chromatin-associated lncRNA whose levels correlate with those of *Fshb* and *Kcna4* mRNA

**Figure 2:**
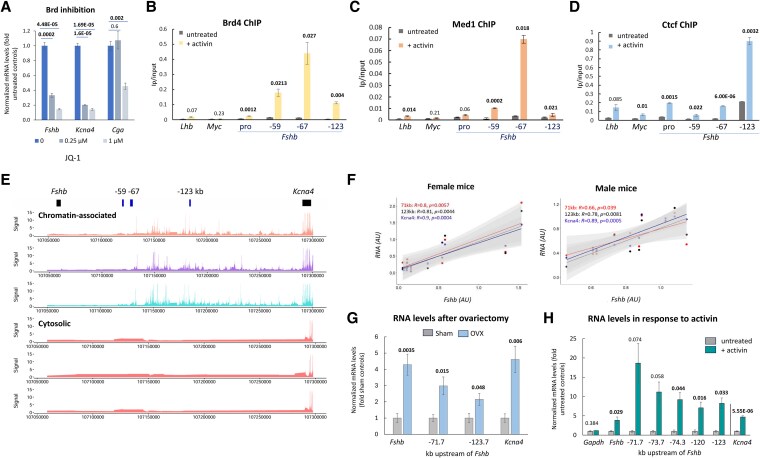
The region has characteristics of a super-enhancer, is associated with chromatin organizational proteins, and transcribed to chromatin-associated lncRNA whose levels correlate with those of *Fshb* and *Kcna4* mRNA. (A) LβT2 cells were exposed to the BET-inhibitor JQ-1 at 0.25 or 1 µM for 24 hours, after which mRNA levels of *Fshb*, *Kcna4,* and *Cga* (as control) were measured by qPCR. Values shown are after normalization to *Rplp0* and as fold those in untreated controls; mean ± SEM, n = 3, *P* values from Student *t*-test compared to untreated controls. (B-D) ChIP was performed for: (B) Brd4, (C) Med1, and (D) Ctcf in control and activin-treated LβT2 gonadotrope cells. Realtime qPCR identified binding at the *Fshb* promoter (pro) and the 3 distal peaks of open chromatin (−59 kb, −67 kb, and −123 kb); *Lhb* and *Myc* serve as controls. IP/input levels are mean ± SEM, n = 3. Student *t*-test compared levels with those in untreated cells at the same locus and *P* values are shown. (E) RNA-seq analysis in LβT2 cells, after separation of chromatin-associated RNA (top 3 panels) and cytosolic RNA (lower 3 panels). (F) Levels of 2 of these lncRNAs (from −71 kb in red and −123 kb in black), and *Kcna4* mRNA (in purple) were measured and compared with those of *Fshb* in the same pituitaries of female and male mice of varying ages; Pearson's correlation coefficient is noted for each. (G) Levels of the same RNAs in sham-operated or ovariectomized (OVX) mice, mean ± SEM, n = 4. Student *t*-test compared mean values with those in sham controls and *P* values are shown. (H) LβT2 gonadotrope cells were treated with activin (5 nM, 24 hours), n = 3 or n = 5 (for *Kcna4*, from a different experiment). Normalized RNA levels are shown relative to mean levels in controls, compared by Student *t*-test as before.

Hypothesizing that this region might function as a super-enhancer, we treated the cells with JQ-1, which represses super-enhancer activity preferentially (10, 40). Accordingly, the treatment reduced dramatically expression of both genes by as much as 10-fold while affecting the control *Cga* much less and only at the higher dose (Fig. 2A). JQ-1 acts by inhibiting Brd4 which, together with Med1, typically marks super-enhancers (7). We thus examined the locus for presence of these proteins, as well as Ctcf, which plays roles in chromatin organization and topology. Chromatin immunoprecipitation for these proteins was performed in a gonadotrope cell line with or without exposure to activin, a major regulator of *Fshb* transcription. The levels of Brd4 increased dramatically at all 3 loci, most notably at −67 kb (Fig. 2B). Med1 levels also increased markedly at the same site following the activin treatment, but less so at −59 kb, and did not change significantly at the more distal site at −123 kb (Fig. 2C). In contrast, Ctcf was most enriched at this distal site, both before and after the activin treatment, which increased its levels very significantly at all 3 loci, and at the *Fshb* promoter (Fig. 2D).

We next looked for any transcriptional activity in the region, and specifically for chromatin-associated RNA (caRNA) which also characterizes enhancers and super-enhancers. Our RNA-seq analysis revealed substantial transcription, particularly between −67 kb and −123 kb, to caRNA that was not detected in the cytoplasm (Fig. 2E). We subsequently used caRNAs transcribed from this region as a read-out for the level of activity of the locus. The levels of these caRNAs, as well as the *Kcna4* mRNA, were positively and significantly correlated with *Fshb* mRNA levels in mice of varying ages in both sexes (Fig. 2F). Moreover, after ovariectomy, not only were the mRNA levels of *Fshb* elevated, as expected, but levels of the lncRNAs and *Kcna4* mRNA also increased (Fig. 2G), further indicating coordinated regulation. We also exposed cultured gonadotrope cells to activin, which increased the level of the lncRNAs in addition to those of *Fshb* and *Kcna4* mRNAs (Fig. 2H). These findings suggest a likely role for the locus in mediating activin-induced *Fshb* expression, and that this region might act as an inducible super-enhancer controlling the differential and coordinated expression of both genes.

### CRISPRi identifies distinct roles of these individual loci in regulating expression of *Fshb* and/or *Kcna4*

**Figure 3:**
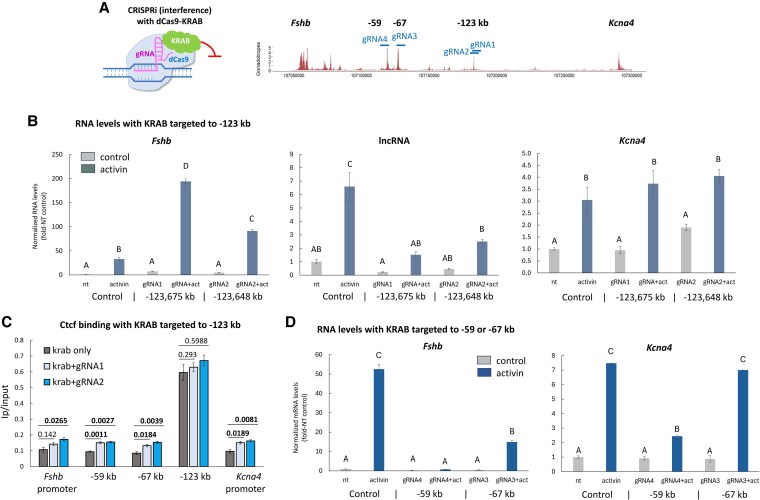
CRISPRi identifies distinct roles of these individual loci in regulating expression of *Fshb* and/or *Kcna4*. (A-D) CRISPRi was performed in LβT2 gonadotrope cells using gRNA targeting dCas9-KRAB to each of the 3 more distal ATAC-seq peaks, at −59, −67, and −123 kb and (B, D) effects on *Fshb* and *Kcna4* mRNAs measured by qPCR. Some of the cells were treated with activin (5 nM for 24 hours), as noted. Normalized levels are presented relative to levels in nontreated controls, mean ± SEM. ANOVA followed by Tukey honestly significant difference post hoc multiple comparisons test was performed, and means sharing the same letter are not significantly different (*P* > .05). (C) ChIP for Ctcf in WT cells and those with dCas-KRAB targeted to −123 kb, performed and presented as in Fig. 2D, n = 3.

To understand the function of each of the individual distal sites in expression of *Fshb* and *Kcna4*, we used CRISPRi, comprising a chimeric dCas9 protein attached to the repressive KRAB domain that was directed to the loci by various gRNAs (Fig. 3A). Surprisingly, targeting this protein to the most distal Ctcf-bound site at −123 kb, increased activin-stimulated mRNA levels of *Fshb*, without affecting those of *Kcna4*, and this despite a drop in the lncRNA levels (Fig. 3B). We hypothesized that this increase in *Fshb* expression might be due to the chimeric protein affecting Ctcf binding and so repeated the Ctcf ChIP in these cells. Although Ctcf binding increased somewhat at the other loci examined, its levels at −123 kb were not altered (Fig. 3C), indicating that this site functions to limit *Fshb* expression though another mechanism that has yet to be determined.

In striking contrast, recruitment of dCas9-KRAB to the −59 and −67 kb sites led to a significant drop in *Fshb* expression, whereas *Kcna4* mRNA levels were reduced only when the construct was targeted to −59 kb (Fig. 3D). Together with the 3C data (Fig. 1E), these results demonstrate that the −67 kb locus regulates *Fshb* selectively, whereas the −59 kb site contributes to the activation of both genes.

### Single-cell multiomics indicates a role for the −67 kb locus during gonadotrope differentiation, possibly mediated by Foxl2

**Figure 4:**
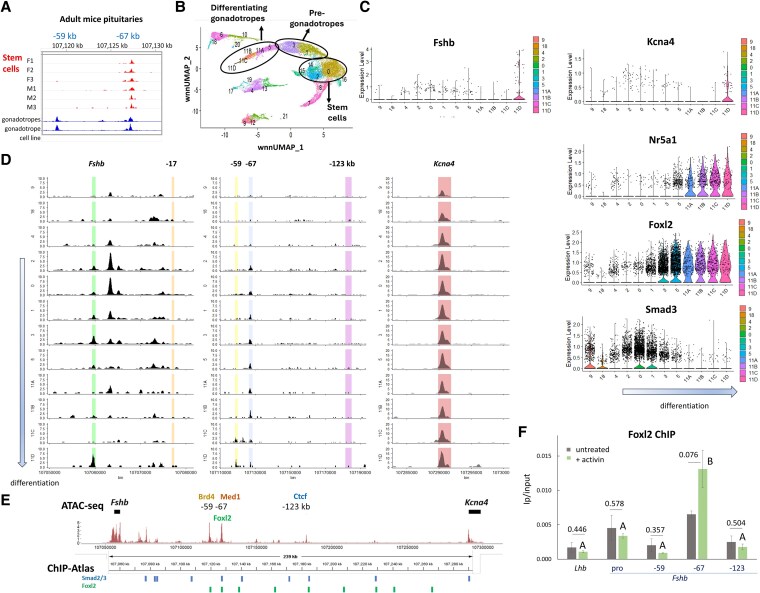
Single cell multiomics indicates a role for the −67 kb locus during gonadotrope differentiation, possibly mediated by Foxl2. (A) Chromatin accessibility at the −59 and −67 kb sites in pituitary stem cells from female (F) and male (M) adult mice (data from (36)), gonadotropes and the αT3-1 gonadotrope precursor cell line (data from (35)). (B–D) Multiomics on neonatal pituitary stem cell-derived cells at postnatal day 3, which differentiate mostly into gonadotropes, represented in the UMAP by the trajectory from the stem cell cluster 2 to the gonadotropes cluster 11, which is further divided into 4 subclusters (11A-D). (C) Expression levels of *Fshb, Kcna4, Nr5a1, Foxl2,* and *Smad3* in some of the cell clusters. (D) The snATAC-seq data at the *Fshb* promoter and upstream sites in the same cell clusters. Highlighted regions include the −59, −67, and −123 kb loci, as well as the −17 kb site and the *Fshb* and *Kcna4* genes. (E) Foxl2 and Smad binding sites in other tissues (from https://chip-atlas.org/), shown aligned with the snATAC-seq data (from Fig. 1B) and protein binding (from Fig. 2B–2D). (F) ChIP for Foxl2 in gonadotropes, analyzed and presented as in Fig. 2, with *t*-test comparisons between levels in treated and nontreated cells, and ANOVA followed by Tukey honestly significant difference between all loci in treated cells (in untreated cells levels did not differ between groups: *P* > .05).

Although the chromatin at these distal sites is closed in the differentiated nongonadotrope cell populations of the adult pituitary (Fig. 1B), the −67 kb site is partially open in the adult pituitary stem cells of both sexes (Fig. 4A). Hypothesizing that this site might be kept open by the binding of a gonadotrope-specific factor expressed early during differentiation, we went on to examine gene expression and chromatin accessibility dynamics at these loci during postnatal gonadotrope differentiation.

To investigate the mechanisms underlying gonadotrope differentiation, we performed single-cell multiomic profiling, at postnatal day 3, of pituitary SOX9iresGFP-positive cells that comprise stem cells and their immediate progeny, given that GFP persists longer than SOX9 (31). At this developmental stage, these cells differentiate almost exclusively to gonadotropes (25). Our approach thus captures coordinated chromatin accessibility and gene expression in the distinct cell clusters, depicting the trajectory of gonadotrope differentiation (Fig. 4B–4D).

Cells in cluster 11, which can be subdivided into 4 subclusters (Fig. 4B), comprise the most differentiated gonadotropes. *Fshb* mRNA is detected only at low levels and in the most differentiated subcluster 11D, remarkably like *Kcna4* (Fig. 4C). Expression of these genes is preceded by that of the lineage-specific markers *Nr5a1* and *Foxl2* (Fig. 4C).

Although the *Kcna4* locus appears accessible in all clusters, the chromatin at the *Fshb* promoter is relatively more open in the 11D subcluster compared with 11A-C (Fig. 4D), consistent with its expression profile. Surprisingly, however, the *Fshb* promoter is partially open in the neonatal stem and early pre-gonadotrope populations (clusters 2, 0, and particularly cluster 3). In these stem and early differentiating clusters, the −67 kb site is also accessible, though closed in the nongonadotrope lineages (Fig. 4D, Dcn+ cluster 9, melanotroph cluster 18, and the Pit1+ cluster 4). The sites at −59 and 123 kb, as well as the previously described −17 kb enhancer, all appear inaccessible at this time, with only the −59 kb site showing some sign of opening in clusters 11C,D.

The accessibility of the −67 locus in the stem and early differentiating cells suggests that a factor maintaining the open chromatin at −67 kb is lost in nongonadotrope populations or is replaced by a gonadotrope-specific factor during lineage commitment. Among transcription factors known to regulate *Fshb* (41-45), Smad3 levels decrease between clusters 1 and 3, whereas Foxl2 levels increase (Fig. 4C); both factors are seen in other tissues to bind the locus, including at the −67 kb site (Fig. 4E). We thus examined Foxl2 binding in untreated or activin-treated LβT2 gonadotrope cells and found it to be significantly enriched at the −67 kb site in the treated cells (Fig. 4F).

### Foxl2 maintains the open chromatin landscape at −67 kb and interacts with the Supt16h histone chaperone

**Figure 5:**
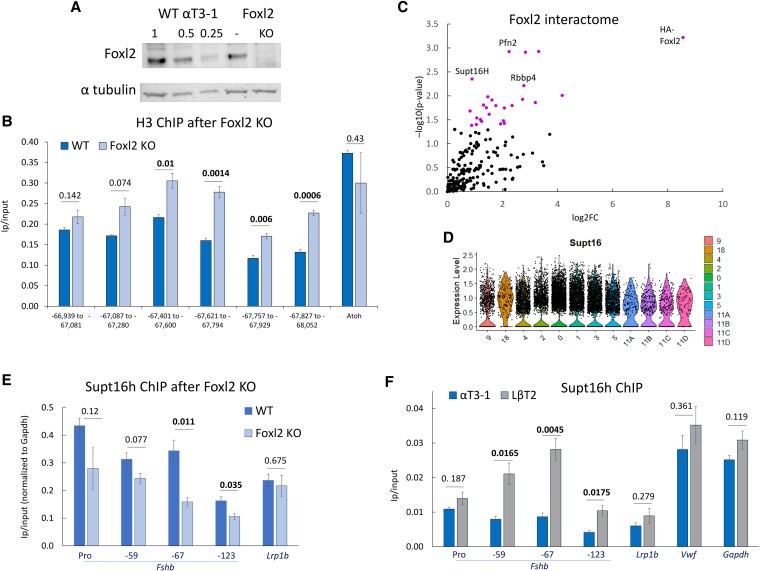
Foxl2 maintains the open chromatin landscape and interacts with the Supt16h histone chaperone. (A) Foxl2 levels in WT αT3-1 cells (loaded at 1, 0.5, or 0.25 quantities) and in the Foxl2 KO cells (lane marked “–” is not relevant for this study). See also Fig. S3 (27). (B) ChIP for H3 in the WT and Foxl2 KO cells, across the −67 kb locus, with *Atoh* as control, presented and analyzed as in Fig. 2. (C) HA-Foxl2 interacting proteins in LβT2 gonadotrope cells (after benzonase treatment), purple dots denote proteins significantly enriched (*P* < .05). (D) Supt16h (Supt16) expression in the same neonatal pituitary cell clusters shown in Fig. 4. (E, F) ChIP for Supt16h in (E) WT αT3-1 and Foxl2 KO cells, shown normalized to levels at *Gapdh* positive control, and (F) also in LβT2 cells, shown as ip/input, as before.

Having established that Foxl2 binds the −67 kb site, we sought next to clarify its role. We knocked out Foxl2 in the αT3-1 gonadotrope-precursor cells via CRISPR editing, leaving the protein undetectable (Fig. 5A). To assess the effect on chromatin compaction at this distal enhancer site, we performed ChIP for histone H3, which revealed that loss of Foxl2 increased H3 levels across this locus (Fig. 5B).

The effect of Foxl2 KO on the chromatin landscape led us to consider that Foxl2 might recruit chromatin remodeling factors to this site. A previous study in αT3-1 cells identified a very large Foxl2-interactome (46), so we performed coimmunoprecipitation of HA-tagged Foxl2 in untreated and activin A-treated (5 nM for 24 hours) LβT2 cells under more stringent conditions using benzonase. This facilitates enrichment for proteins interacting directly with Foxl2 rather than via nucleic acids. The precipitated proteins included 45 factors that were not present in any of the controls. Of these, only Rps21 and Rrbp1 were significantly affected by the activin treatment (*P* < .05) and were detected only in the untreated samples. We therefore pooled treated and untreated HA-Foxl2 IP samples and compared their intensities against the pooled controls. The proteins most significantly enriched and not present in controls comprised Pfn2, Supt16h, and Rbbp4 (*P* < .05: Fig. 5C). Supt16h was among Foxl2-interacting proteins detected in the previous study in αT3-1 cells, and the interaction was confirmed in that study by western blot (46). Furthermore, analysis of ChIP-seq data in the ChIP-atlas revealed that of 3356 binding sites detected for Supt16h, 35% (1187) intersect with Foxl2 binding, and 14% (472) also with Rbbp4.

Supt16h (Supt16) is a component of the FACT histone chaperone complex and therefore appeared particularly interesting and potentially relevant in shaping the chromatin landscape at the *Fshb* enhancer. It is expressed abundantly in the neonatal pituitary stem cells and those differentiating to gonadotropes (Fig. 5D). We thus performed ChIP for Supt16h initially in the WT or Foxl2 KO αT3-1 cells. The Foxl2 KO more than halved Supt16h binding at the −67 kb site and it was also reduced at the −123 kb locus (Fig. 5E). Supt16h was detected at the −59 kb and *Fshb* promoter, though this was not significantly affected by the Foxl2 KO. We also examined Supt16h binding in LβT2 cells, in which the *Fshb* gene is expressed, and compared its levels with those in the αT3-1 cells in which it is not expressed. ChIP confirmed that Supt16h association with all 3 distal sites of the super-enhancer region was considerably greater than in the αT3-1 cells (Fig. 5F), confirming a positive correlation with *Fshb* expression levels.

## Discussion

Our study identifies a distal regulatory domain upstream of *Fshb* that functions as a super-enhancer governing transcription across the *Fshb-Kcna4* locus, with Foxl2 playing a role in shaping its chromatin landscape. This builds on our previous studies of the *Cga* and *Lhb* loci, where individual distal elements modulate transcription through eRNA production and chromatin remodeling (5, 21). Here, however, we reveal a more complex system, with roles not only in *Fshb* transcription but also in gonadotrope lineage specification and, surprisingly, coordinated expression of *Kcna4*. Although a role for the Kcna4 potassium voltage-gated channel in the pituitary gonadotrope has not been reported, other potassium channels function in the release of various reproductive hormones (47), and *Kcna4* expression changes through the estrous cycle (48). Notably, upstream of the *Lhb* gene lies another potassium channel gene, *Kcna7*, which is expressed in LβT2 gonadotrope cells, raising the possibility that potassium channels contribute more broadly to pituitary secretory dynamics and that Kcna4 may participate in FSH secretion.

The presence of several enhancer elements in the gene desert upstream of *Fshb* is in accordance with other lineage-defining loci (49-51) and aligns with the concept that super-enhancers contain multiple distinct regulatory modules (52). The 3 enhancer elements that we studied here share common features of gonadotrope-specific open chromatin and recruitment of chromatin-organizational proteins (53), and the region is transcribed abundantly to caRNA. These findings suggest possible roles in the formation of chromatin-associated condensates that reportedly assemble at transcriptional hubs and might contribute to looping, factor recruitment, or activation competence (7, 54-57). Responsiveness of these proteins and caRNAs to activin and/or ovariectomy could well facilitate the large increases in *Fshb* transcription that occurs after ovariectomy or during perimenopause, during which activin stimulation of *Fshb* is markedly elevated (58, 59).

Although seeming to form a regulatory domain, these elements have distinct features that differentially affect basal and/or activin-stimulated *Fshb* and *Kcna4* expression. The −67 kb site appears central to gonadotrope lineage acquisition and *Fshb* expression, whereas the −59 kb element engages later in gonadotrope differentiation and integrates activin signaling to modulate both *Fshb* and *Kcna4*. The role of the −59 kb site in regulating *Kcna4* expression is notable, given that *Kcna4* is also expressed in corticotropes where this element is not accessible, suggesting that it functions as a cell-type-specific enhancer for this gene. The −123 kb region behaves differently, as its repression increased expression of both genes even as caRNA levels dropped. The mechanisms underlying this repression, including the role of Ctcf and other factors, will require further study, but these findings do indicate that the caRNA does not mediate these stimulatory responses directly.

By combining chromatin accessibility with transcriptional trajectories, we have moved beyond static snapshots to a map of enhancer engagement over time, to begin to understand how signaling and lineage cues are integrated at this locus. That the −67 kb element is at least partially accessible in the pituitary stem cells suggests that it is already primed and highlights enhancer gating as a determinant of lineage identity. Foxl2 is well recognized for its roles in *Fshb* promoter activation in mature gonadotropes, though it is one of the earliest markers of the gonadotrope-lineage and expressed well before *Fshb*. Its role here is consistent with a model in which Foxl2 contributes to maintaining chromatin accessibility at this site as differentiation proceeds, whereas in other pituitary lineages the region becomes closed. This function may involve recruitment of Supt16h of the FACT complex, linking Foxl2 to nucleosome turnover and active chromatin remodeling. More broadly, these findings highlight transcription factors as key architects of accessibility during pituitary cell-fate acquisition.

At the start of this study, no role had been described for this extensive region in *Fshb* regulation, though the −17 kb site has since been reported as an enhancer for this gene (22-24). Moreover, at the time of this submission, a new study (60) reports the limited activity of this −17 kb site, as well as activity of the −59 and −67 kb sites in reporter assays. They also provide extensive evidence in LβT2 cells for enrichment of Foxl2 and Smad2/3 binding, and of H3K27ac at this locus. Although taking a different approach, their findings complement ours and highlight the −59 and −67 kb sites as likely important regulators of *Fshb* transcription.

In summary, this work defines a gonadotrope-specific super-enhancer that shows Foxl2-dependent maintenance of chromatin accessibility across the *Fshb-Kcna4* locus, establishes enhancer priming as an early feature of gonadotrope differentiation, and illustrates how modular enhancer architecture supports coordinated and gene-specific regulation. These insights expand our knowledge of how pituitary gene expression is determined by distal chromatin states and provide a basis for understanding how hormonal signals intersect with the chromatin to regulate *Fshb* expression. They will also facilitate interpretation of the effects of noncoding variation associated with human reproductive disorders.

## Data Availability

Original caRNA-seq datasets are available at GEO, NCBI (GSE312515), mass spectrometry proteomic datasets are at ProteomeXchange Consortium via the PRIDE partner repository with the dataset identifier PXD072033, and multiomics datasets are available at GEO, NCBI (GSE315967). Other original data analyzed in this study are in the data repositories listed in the References, newly generated data included in the manuscript, or available from the corresponding author on reasonable request.

## Abbreviations

3C: chromatin conformation capture
caRNA: chromatin-associated RNA
ChIP: chromatin immunoprecipitation
eRNA: enhancer RNA
lncRNA: long noncoding RNA
qPCR: quantitative PCR
TAD: topologically associated domain
TSS: transcriptional start site
UMAP: uniform manifold approximation and projection
WT: wild-type
